# Interference experiment with asymmetric double slit by using 1.2-MV field emission transmission electron microscope

**DOI:** 10.1038/s41598-018-19380-4

**Published:** 2018-01-17

**Authors:** Ken Harada, Tetsuya Akashi, Kodai Niitsu, Keiko Shimada, Yoshimasa A. Ono, Daisuke Shindo, Hiroyuki Shinada, Shigeo Mori

**Affiliations:** 1 CEMS, RIKEN (The Institute of Physical and Chemical Research), Hatoyama, Saitama 350-0395 Japan; 2 Research & Development Group, Hitachi, Ltd., Hatoyama, Saitama 350-0395 Japan; 3 Department of Materials Science, Osaka Prefecture University, Sakai, Osaka 599-8531 Japan; 4 Department of Applied Quantum Physics and Nuclear Engineering, Kyushu University, Fukuoka, Fukuoka 819-0395 Japan; 5 IMRAM, Tohoku University, Sendai, Miyagi 980-8577 Japan

## Abstract

Advanced electron microscopy technologies have made it possible to perform precise double-slit interference experiments. We used a 1.2-MV field emission electron microscope providing coherent electron waves and a direct detection camera system enabling single-electron detections at a sub-second exposure time. We developed a method to perform the interference experiment by using an asymmetric double-slit fabricated by a focused ion beam instrument and by operating the microscope under a “pre-Fraunhofer” condition, different from the Fraunhofer condition of conventional double-slit experiments. Here, pre-Fraunhofer condition means that each single-slit observation was performed under the Fraunhofer condition, while the double-slit observations were performed under the Fresnel condition. The interference experiments with each single slit and with the asymmetric double slit were carried out under two different electron dose conditions: high-dose for calculation of electron probability distribution and low-dose for each single electron distribution. Finally, we exemplified the distribution of single electrons by color-coding according to the above three types of experiments as a composite image.

## Introduction

Recent state-of-the-art technologies on electron microscopes (EMs) have provided us an advanced EM system equipped with generation of coherent electron beams, their precise operations during the propagation, and single electron detection. When spatial spreads of existence probabilities of electrons, which depend on Heisenberg’s uncertainty principle, become larger than the double-slit size, it becomes possible to precisely carry out double slit experiments, which indicate wave-particle duality of electrons. This wave-particle duality is one of the fundamental properties of fine particles, for example, electrons, subject to quantum physics. It was pointed out by Feynman^1^ as one of the mysteries of quantum physics. Various experimental observations have been reported on the wave-particle duality of electrons^2–11^; however, this mysterious issue has not been fully resolved yet.

In 1961 Jönsson reported the first wave-particle duality experiment using 50-keV electron beams with a special technique for fabricating thin single-, double-, and multi-slits^2,3^. Subsequent double-slit experiments were reported by Merli *et al*. in 1976^4^, Lichte in 1988^5^, and Tonomura *et al*. in 1989^6^ using electron biprisms (BPs)^12^ instead of double slits. They succeeded in observing single electrons forming interference fringes, i.e., single-electron buildup. Double-slit experiments have also been carried out on photons^13–15^, atoms^16^, and molecules^17^. In particular, single photon trajectories in a double-slit interferometer were discussed^14^. All these experiments attempted to observe wave-particle duality.

Recent technological developments have made it possible to perform double-slit experiments more easily. These technologies include focused ion beam (FIB) instruments for fabricating thin slits^7–11^ and fast-readout pixel detectors^10^ for detailed and precise observations of single-electron stacking and fringe formation. Almost all of the modern interference experiments on electrons using EMs^7–11^, however, used thermal-emission electrons, which caused the following ambiguity: whether an electron can simultaneously passed through double slits as a coherent beam. Furthermore, most of these experiments used symmetric double slits and were conducted under the Fraunhofer conditions for the double slit; i.e., electron beams were superposed far away from both slits, resulting in losing information on which slit a particular electron passed through. In two of the experiments^9,11^ asymmetric double slit were used, but no identification was reported regarding which slit the electrons passed through.

In the present double-slit experiments, we used the following technologies and methods to clarify these ambiguities: (1) highly coherent electron beams from a recently developed 1.2-MV field-emission transmission electron microscope (FE-TEM)^18,19^, (2) asymmetric double slits having slit widths that can be varied by using a biprism, and (3) measurements under a pre-Fraunhofer condition at distances shorter than those in the Fraunhofer condition. Here, the pre-Fraunhofer condition indicates that for single-slit observations, the distance between the double slit and the image plane is sufficiently long to be considered as a Fraunhofer condition, while for the double-slit observations, this distance is too short to be considered as a Fraunhofer condition. The pre-Fraunhofer condition can be described by the equation ${d}_{w}^{2}/\lambda \ll {\rm{\Delta }}f\ll {d}_{s}^{2}/\lambda$, where Δ*f* is the distance from the double slit to the image plane representing a defocus length, *d*_w_ is the width of the slit opening, *d*_s_ is the spacing between the slits, and *λ* is the wavelength of the electron beam. Electrons passing through each asymmetric slit form different Fraunhofer patterns at the image plane, while electrons passing through the double slit (both slits simultaneously) form two-wave interference fringes between these Fraunhofer patterns. We used these three interference patterns to distinguish three types of electron: electrons passing through only one of the asymmetric slit; electrons passing through both slits simultaneously; and electrons passing through only the other slit.

In this letter we report confirmation of “single-electron buildup” fringes by using symmetric nano-sized double slits under the Fraunhofer condition and an interference experiment using an asymmetric double slits realized by using one of the electron BPs under the pre-Fraunhofer condition to obtain a clear result for which slit electrons passed through.

## Results

**Figure 1:**
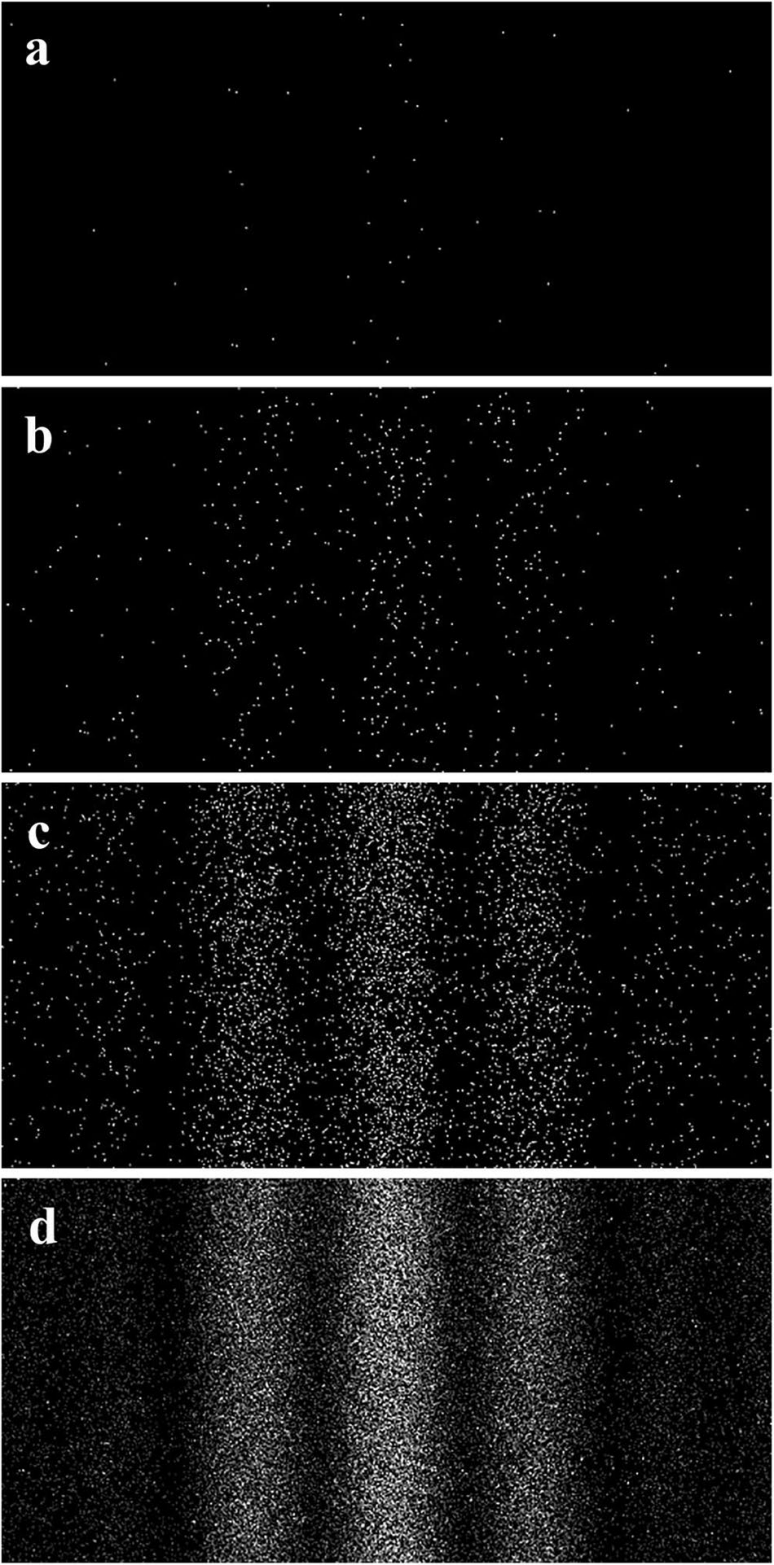
Fringes observed in a single-electron-buildup experiment in which the electron dose is controlled by the exposure time: (**a**) approximately 50 electrons were counted in the panel in 0.1 s in the viewing area, (**b**) approximately 500 electrons in 1.0 s, (**c**) approximately 5,000 electrons in 10 s, and (**d**) approximately 50,000 electrons in 100 s.

Figure 1 shows an example of the interference fringe formation process, i.e., a “single electron buildup” experiment, under a very low dose condition for the incident electron beam intensity. The electron count rate on the detector plane was stable at 0.001 electrons/pixels/s during the buildup experiment. The average distance between two consecutive propagating electrons was estimated to be more than 2,500 m under the condition of 4,200 times magnification and the irradiation area of 100 μm in diameter on the double-slit plane. Then the divergence angle of the incident electron beam to the double slit was less than 10^–7^ rad, leading to a spatial coherence length of more than 10 μm^20^, which satisfies the “single electron buildup” condition.

Even at this low dose image, many electrons were detected at positions of interference fringes, indicating little interference of shot noises on images. This analysis confirms that the camera system on the microscope can detect single electron positions.

### Asymmetric Double-Slit

**Figure 2:**
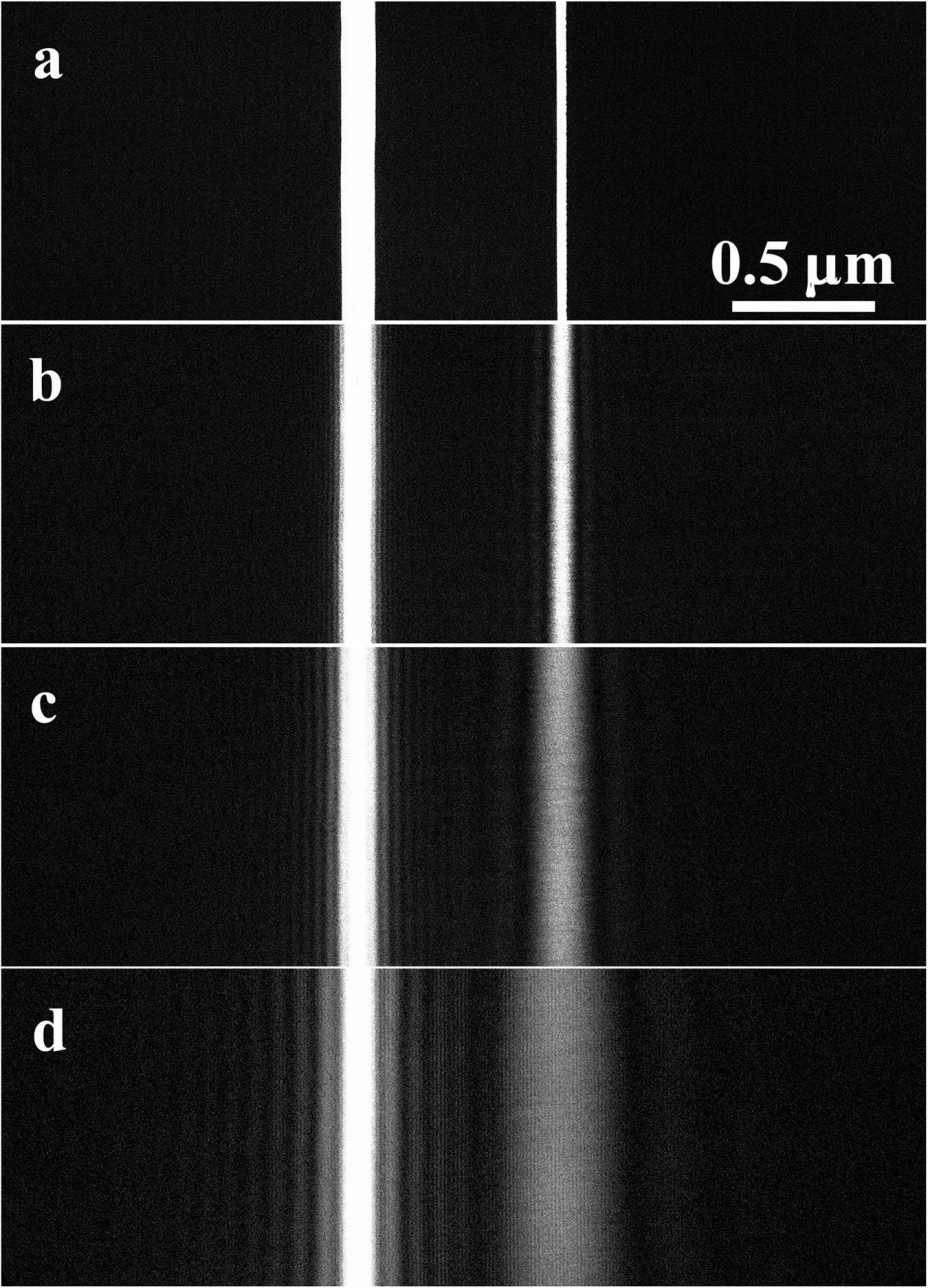
Through-focus series of an asymmetric double slit, whose rectangle openings are of 0.12 μm width for the left slit and 0.035 μm width for the right slit, with a 0.8 μm spacing between the slits *d*_*s*_: (**a**) in-focus condition (Δ*f* = 0 mm), (**b**) Fresnel condition #1 for defocusing Δ*f* = 4 mm estimated from the lens current, (**c**) Fresnel condition #2 for defocusing Δ*f* = 7 mm and interference-fringe spacing *s* = 8 nm, and (**d**) pre-Fraunhofer condition for Δ*f* = 16 mm and *s* = 15 nm. In Fig. 2(d), the images of each single slit show Fraunhofer patterns but they do not overlap; therefore, the image of the double slit is under the Fresnel condition. However, two-wave interference fringes between the Fraunhofer patterns are observed as in Fig. 3(b-2).

In order to clarify which slit electrons passed through under the buildup condition, we used an asymmetric double-slit and carried out the interference experiment under the pre-Fraunhofer condition. Figure 2 shows a through focus series obtained by using the asymmetric double slit: (a) in-focus condition, (b) Fresnel condition #1; (c) Fresnel condition #2; and (d) pre-Fraunhofer condition. Here, the width of the right slit was narrowed down to 0.035 μm from 0.12 μm by partially covering the right end of the slit with the first BP filament. We note that the images for the wider slit need a longer defocusing length to reach the Fraunhofer condition. In the single-slit experiment, each single slit pattern in Fig. 2(d) was recorded under the Fraunhofer condition, while for the double slit experiment, the double slit pattern was recorded under the Fresnel condition. To describe these experimental conditions, we named these conditions collectively the pre-Fraunhofer condition. In Fig. 2(c) and (d), two-wave interference fringes with a spacing *s* were observed in the area between the slits. The defocus length Δ*f* is given by ${\rm{\Delta }}f=({d}_{s}/\lambda )\times s$.

To obtain information on the fringe formation in the present double-slit interference experiment, we focused on the fringes for the asymmetric double slit under the pre-Fraunhofer condition shown in Fig. 2(d), and particularly we focused on experimentally finding out which slit electrons passed through under a low dose condition, such as 0.02 electrons/pixel on average. Then we were able to observe single electron behavior at a time because an averaged distance between two consecutive propagating electrons became approximately 125 m, long enough to eliminate electron correlation.

### Low-dose/high-dose observation

To find out probability distribution of electron existence that passed through the asymmetric slit, three interference patterns were recorded: patterns for electrons passed through each single slit and a pattern for electrons passed through the double slit. Two electron dose conditions for interference recordings were used: high electron dose of 20 electrons/pixel for obtaining continuous electron probability distribution and low electron dose of 0.02 electrons/pixel for obtaining single electron distribution.

**Figure 3:**
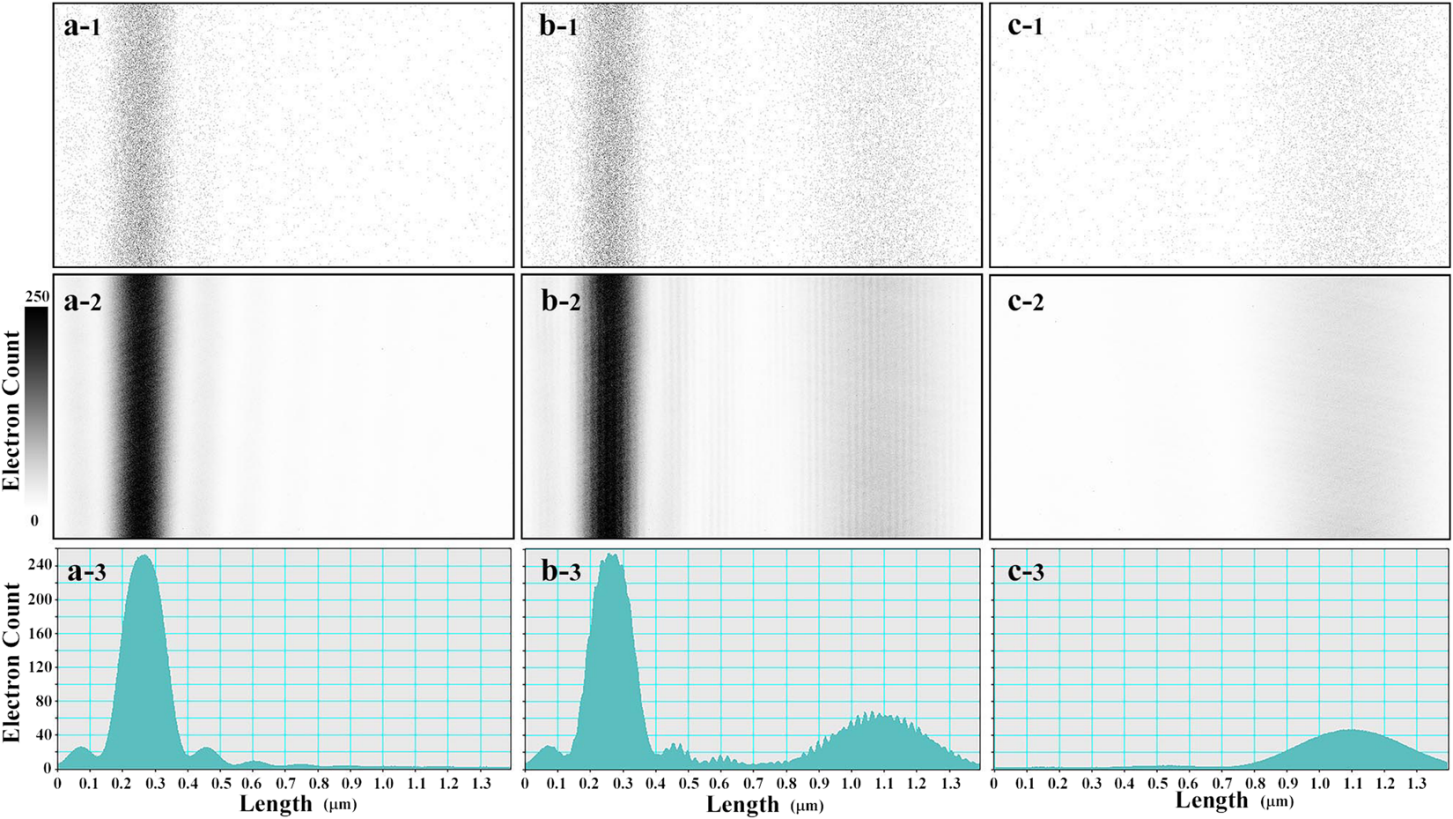
Pre-Fraunhofer patterns for defocusing of Δ*f* = 22 mm and their intensity profiles: (**a**) electrons that passed through only the left slit, (**b**) electrons that passed through the double slit, and (**c**) electrons that passed through only the right slit. Patterns in the top row (a-1), (b-1), and (c-1) are for the pre-Fraunhofer patterns at a low electron dose of 0.02 electrons/pixel on average. Patterns in the middle row (a-2), (b-2), and (c-2) are for a relatively high electron dose of 20 electrons/pixel on average. Patterns in the bottom low (a-3), (b-3), and (c-3) are averaged intensity profiles. Contrasts of the Fraunhofer patterns are reversed for a better view.

Figure 3 shows the results for electron interferograms (pre-Fraunhofer patterns) and intensity profiles. Figure 3(a-1),(a-2), and (a-3) show data for only the left slit open; Figure 3(b-1),(b-2), and (b-3) show data for the asymmetric double-slit experiment; Fig. 3(c-1),(c-2), and (c-3) show data for only the right slit open. For clear image representation, contrasts of the interferograms are reversed; the single electrons are indicated as sparse black dots. Figure 3(a-1),(b-1), and (c-1) show low dose patterns with 0.02 electrons/pixel at a 0.1 s exposure, and each dot in the images corresponds to each single electron; Fig. 3(a-2),(b-2), and (c-2) show relatively high dose patterns with 20 electrons/pixel at a 100 s exposure; Fig. 3(a-3),(b-3), and (c-3) denote averaged intensity profiles of the patterns in Fig. 3(a-2),(b-2), and (c-2), respectively. We note that the high dose pattern in Fig. 3(b-2) shows two-wave interference fringes for the double-slit experiment.

## Discussion

### Simulation of asymmetric double slit experiment

**Figure 4:**
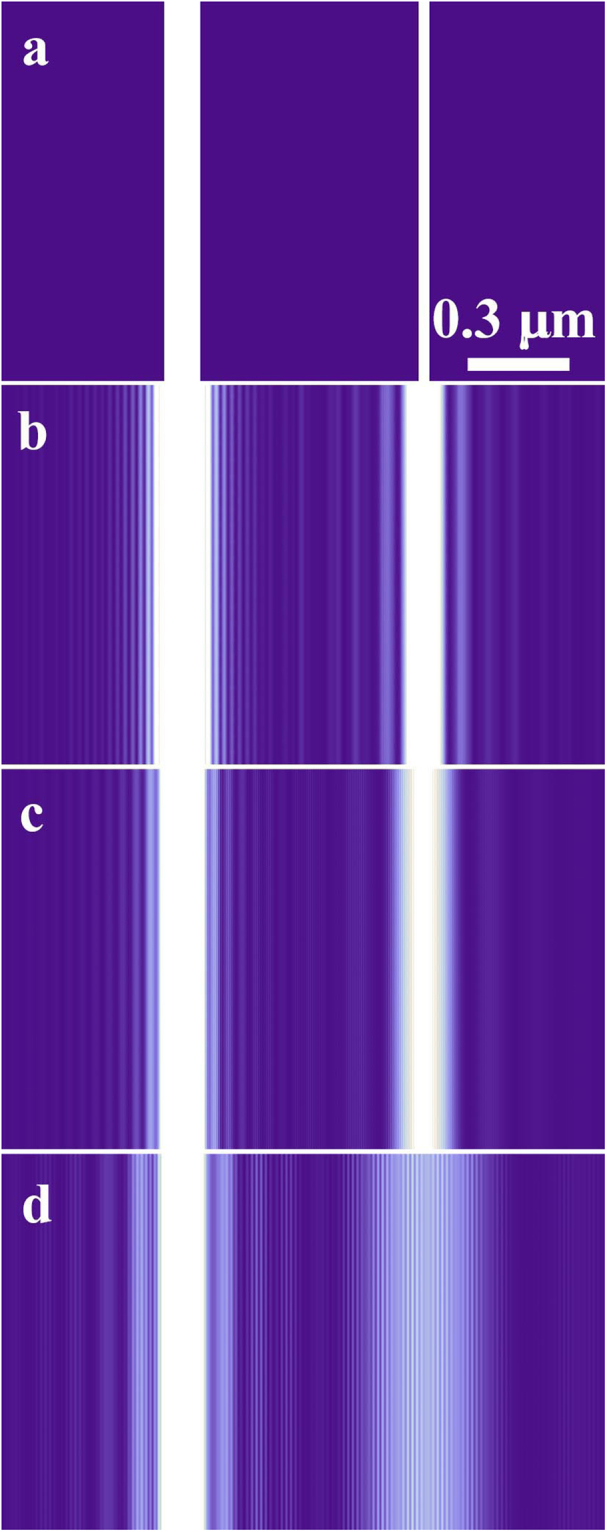
Results of numerical calculation depending on wave optics reflecting the interference experiment of the asymmetric double slit shown in Fig. 2: (**a**) in-focus condition (Δ*f* = 0 mm), (**b**) Fresnel condition #1 for defocusing Δ*f* = 4 mm, (**c**) Fresnel condition #2 for defocusing Δ*f* = 7 mm, and (**d**) pre-Fraunhofer condition for Δ*f* = 16 mm.

Figure 4 shows interference fringe patterns numerically calculated based on the Fresnel diffraction in wave optics^21^. Values for parameters in the calculation are taken to be equal to those of Fig. 2. The interference patterns in Fig. 4 are in good agreement with those of Fig. 2, reflecting wave characteristic of electrons even in this asymmetric double slit experiment, i.e., electrons must have passed through both slits as the same time.

### Two-wave interferometry analysis

The results of the double slit experiment can be explained in terms of interference of two waves. We define the electron wave that pass through the left slit as |*φ*_left_> and the electron wave that pass through the right slit as |*φ*_right_>. Then they can be written as

1 $$|{\phi }_{{\rm{left}}}\rangle ={\varphi }_{{\rm{left}}}\exp (i{\eta }_{{\rm{left}}}),$$

2 $$|{\phi }_{{\rm{right}}}\rangle ={\varphi }_{{\rm{right}}}\exp (i{\eta }_{{\rm{right}}}),$$

where *ϕ*_left_ and *ϕ*_right_ are the amplitude distributions and *η*_left_ and *η*_right_ are the phase distributions of the electron waves at the image plane after passing through each of the single slits. The intensity distribution of the pre-Fraunhofer interference patterns *I*_interfer_ can be described in the two-wave interferometry analysis as

3 $$\begin{array}{rcl}{I}_{{\rm{interfer}}} & = & \langle {\phi }_{{\rm{left}}}|{\phi }_{{\rm{right}}}\rangle ={|{\varphi }_{{\rm{left}}}|}^{2}+{|{\varphi }_{{\rm{right}}}|}^{2}+2|{\varphi }_{{\rm{left}}}||{\varphi }_{{\rm{right}}}|\cos \,({\eta }_{{\rm{right}}}-{\eta }_{{\rm{left}}})\\  & = & {{\rm{\Phi }}}_{{\rm{left}}}+{{\rm{\Phi }}}_{{\rm{right}}}+{{\rm{\Phi }}}_{{\rm{both}}}\end{array}$$

where, ${{\rm{\Phi }}}_{{\rm{left}}}=\langle {\phi }_{{\rm{left}}}|{\phi }_{{\rm{left}}}\rangle ={|{\varphi }_{{\rm{left}}}|}^{2}$ is the intensity distribution of the electron wave that passes through only the left slit, ${\Phi }_{{\rm{right}}}=\langle {\phi }_{{\rm{right}}}|{\phi }_{{\rm{right}}}\rangle ={|{\varphi }_{{\rm{right}}}|}^{2}$ is the intensity distribution of the electron wave that passes through only the right slit, and Φ_both_ is the intensity distribution of the electron wave that passes through both slits simultaneously. The cosine function in the third term of Eq. (3) represents the two-wave interference fringes.

The intensity distributions of electrons in Fig. 3 can be considered to be probability distributions at the image plane where electrons reach. Figure 3(a-2),(b-2), and (c-2) correspond to Φ_left_, Φ_both_, and Φ_right_, respectively. In particular, Φ_both_, the intensity distribution of the interfered electrons that passed through both slits simultaneously, is given by

4 $${{\rm{\Phi }}}_{{\rm{both}}}={I}_{{\rm{interfer}}}-{{\rm{\Phi }}}_{{\rm{left}}}-{{\rm{\Phi }}}_{{\rm{right}}}=2\sqrt{{{\rm{\Phi }}}_{{\rm{left}}}}\sqrt{{{\rm{\Phi }}}_{{\rm{right}}}}\,\cos ({\eta }_{{\rm{right}}}-{\eta }_{{\rm{left}}}).$$

In the following argument, we extracted electrons described by Φ_both_ from the interference patterns in Fig. 3 and showed that we can categorize electrons in three groups: electrons passed through each single slits and electrons passed through both slits at the same time. For that purpose, we used high dose patterns shown in Fig. 3(a-2),(b-2), and (c-2) to estimate probability distributions, and we used low dose patterns in Fig. 3(a-1),(b-1), and (c-1) as directly measured data of individual electrons.

**Figure 5:**
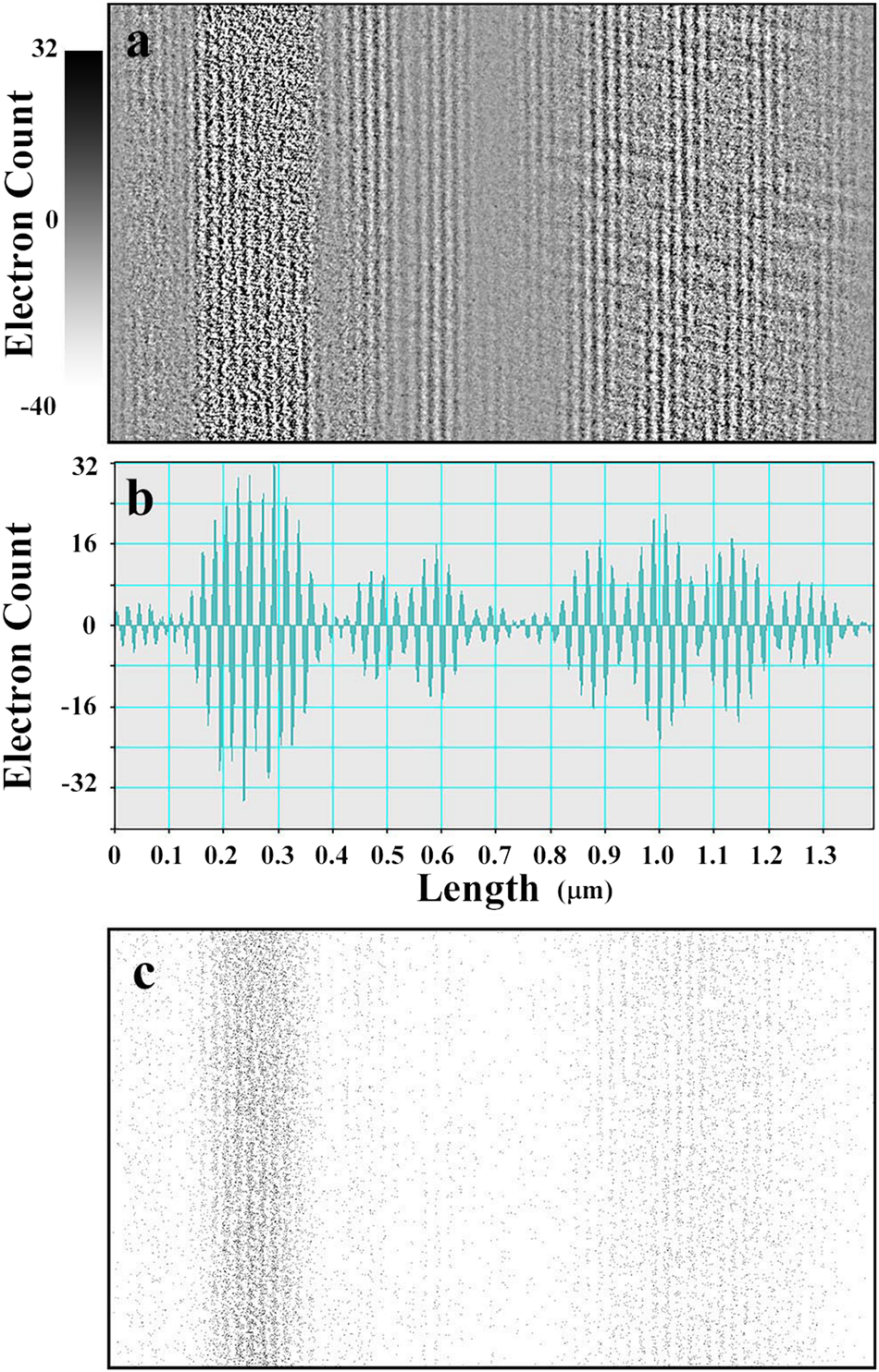
(**a**) Pattern of the intensity distribution Φ_both_ obtained by subtracting the data in Fig. 3(a-2),(c-2) from the data in Fig. 3(b-2); (**b**) intensity profile of the above pattern, including negative-valued data; (**c**) extracted low dose interference distribution for electrons passed through both slits simultaneously calculated by multiplying the data of Fig. 3(b-1) with the data of Fig. 5(a). To perform image processing in an ordinary procedure, the calculated negative-valued data in (b) were offset to zero.

Figure 5(a) shows the intensity distribution pattern Φ_both_. Since the background intensity distributions around the Fraunhofer patterns due to single slits are already subtracted from *I*_interfer_, only the two-wave interference fringes from the double slit are shown. Figure 5(b) shows the intensity profile of the subtracted distribution. To perform ordinary image processing in real and positive numbers in intensity distributions, calculated negative-valued data were offset to zero. Figure 5(c) shows the electron distributions extracted from Figs. 3(b-1) (low dose data) corresponding Eq. (4).

### Categorization of electrons

**Figure 6:**
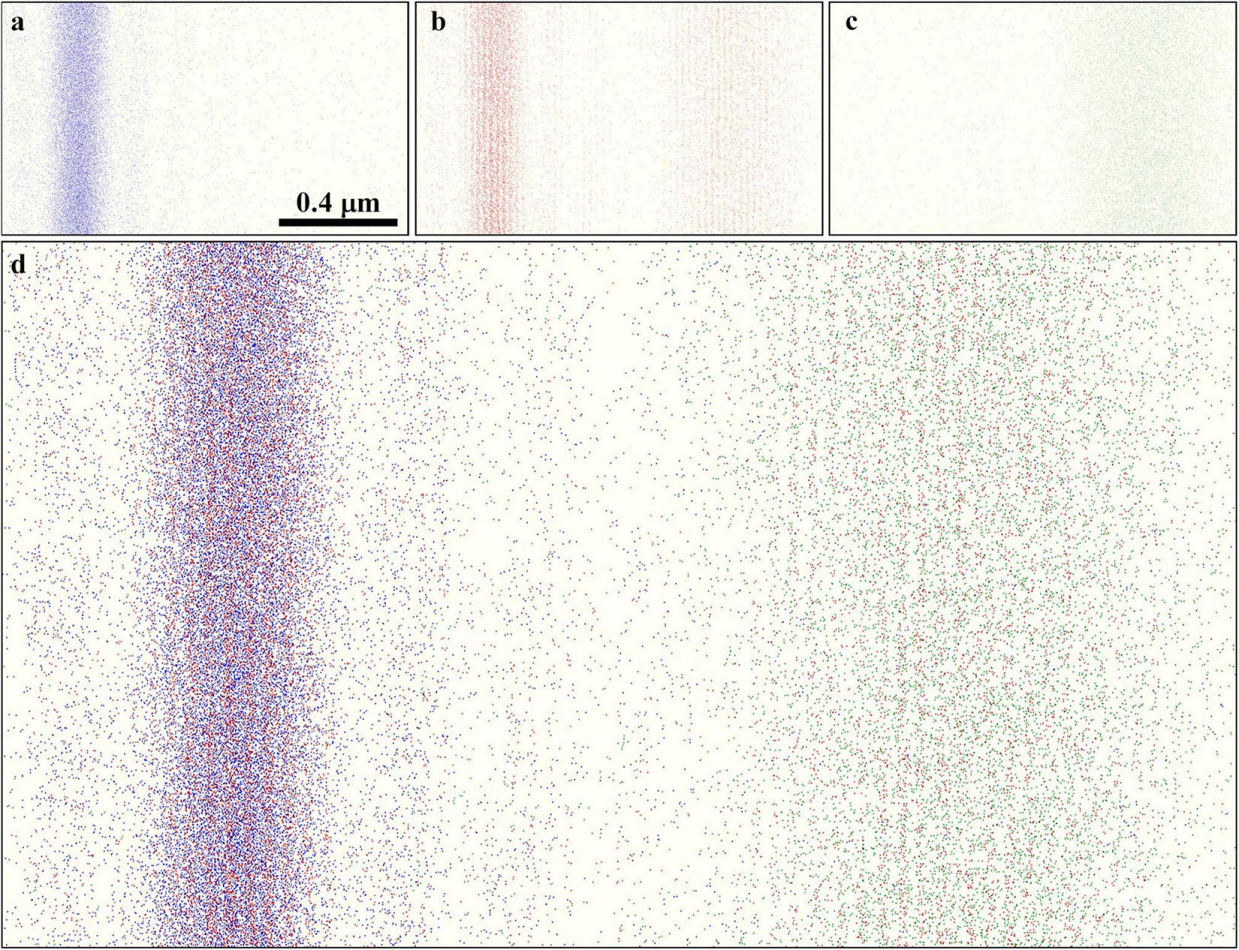
Color-coded electron images from Figs 3(a-1),(c-1), and 5(c). (**a**) Blue colored electrons passed through only the left slit; (**b**) red colored electrons passed though both slits simultaneously; (**c**) green colored electrons passed through only the right slit; and (**d**) composite image of (**a**), (**b**) and (**c**).

Figure 6(a),(b) and (c) show low dose electrons categorized by three colors: blue corresponds to electrons passed through only the left slit; green corresponds to electrons passed through only the right slit; and red corresponds to electrons passed through both slits simultaneously (see, Fig. 5(c)). A composite image of these color-coded electrons is shown in Fig. 6(d), where total intensity might be higher than that of Fig. 3(b-1) because it does not contain negative-valued intensities in the processed interference fringe pattern.

Since Fig. 6(d) is a composite image of three different experimental states, the coherence of the electron wave cannot be discussed directly. If electrons can be categorized, however, it should be distributed as shown in Fig. 6(d). This is because Fig. 6(d) is an experimental result embodying Eq. (4).

## Conclusion

We succeeded in performing double slit experiment by using coherent electron waves and a direct detection camera system of the 1.2-MV FE-TEM. An asymmetric double slit was realized by using a biprism filament for partially masking one of two symmetric slits fabricated by an FIB instrument and by introducing the “pre-Fraunhofer” condition. In the present double-slit experiment, we managed to explicitly categorize electrons that passed through the asymmetric double slits into three types: those that passed through only the left slit; those that passed through both slits simultaneously; those that passed through only the right slit. The intensity profiles of these three types of electron were explicitly shown in color-coded figures.

## Method

We used the 1.2-MV FE-TEM^18,19^ equipped with seven imaging lenses, four BPs for flexibility in electron interferometry, and a direct detection camera system for recording single electrons at a sub-second exposure time.

**Figure 7:**
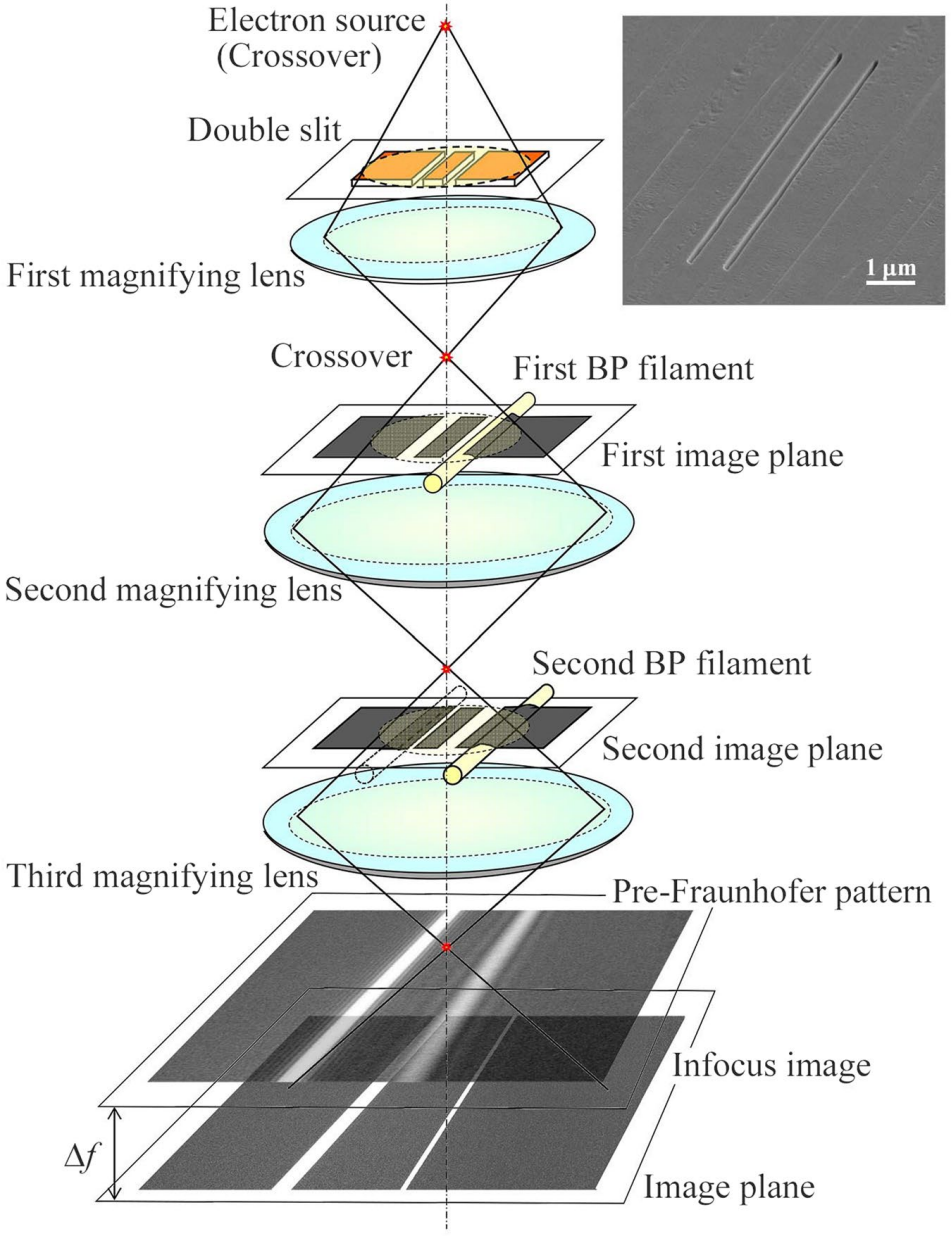
Schematic diagram of optical system. The double slit is placed at the specimen position and is imaged on the first and second electron BP filaments. The double slit and two filaments are in-focused on the image plane in the conjugate relation. The defocusing conditions are controlled by the third magnifying lens. The width of one of the slits is controlled by using the first BP filament and alternation between single-slit and double-slit experiments is carried out by using the second BP filament. The inset shows a scanning electron micrograph of the fabricated double slit, having a 0.12 μm width, 10 μm length, and 0.8 μm spacing between the slits.

Figure 7 shows a schematic diagram of the optical system for the double-slit experiment. A symmetric double slit was placed at the specimen position. The first image of the double slit was formed on the first BP filament between the first and second magnifying lenses. This filament (1.9 μm in diameter) was made of a quartz fiber with Pt-Pd coating and was utilized to control the opening width of one of the slits. The second image of the double slit was formed on the second BP filament between the second and third magnifying lenses. This BP filament (1.6 μm in diameter) was used to shield the beam going through one of the single slits for the alternation between single-slit and double-slit experiments. The double slit and the first and second BP filaments were placed on the conjugate planes and were used to superimpose electron waves as in-focus images^22^. The defocused image patterns formed by changing the third magnifying lens from the in-focus condition to the Fraunhofer condition, and were enlarged by using three successive magnifying lenses.

The double slits consisting of two rectangular openings 0.12 μm in width and 10 μm in length and spaced 0.8 μm apart were made of copper foil 1 μm thick by using an FIB instrument (NB-5000, Hitachi High-Technologies Corp.). Since the slit width of 0.12 μm and the spacing of 0.8 μm were about five orders of magnitudes longer than 0.76 pm, the wavelength of the 1.2-MeV electron beam, the Fraunhofer condition can be satisfied for the double slit under the conditions of a defocusing length of 22 mm or longer and the camera length of 115 m or longer.

The advanced direct detection camera system (3711 × 3839 pixels, K2^®^ Summit Camera, Gatan Co.) was used in electron counting mode.
